# Adjoint traveltime tomography unravels a scenario of horizontal mantle flow beneath the North China craton

**DOI:** 10.1038/s41598-021-92048-8

**Published:** 2021-06-15

**Authors:** Xingpeng Dong, Dinghui Yang, Fenglin Niu, Shaolin Liu, Ping Tong

**Affiliations:** 1 Department of Mathematical Sciences, Tsinghua University, Beijing, 100084 China; 2 Department of Earth, Environmental and Planetary Sciences, Rice University, Houston, TX USA; 3 State Key Laboratory of Petroleum Resources and Prospecting, and Unconventional Petroleum Research Institute, China University of Petroleum at Beijing, Beijing, China; 4 Division of Mathematical Sciences, School of Physical and Mathematical Sciences and Asian School of the Environment, Nanyang Technological University, Singapore, Singapore

**Keywords:** Geophysics, Seismology

## Abstract

The North China craton (NCC) was dominated by tectonic extension from late Cretaceous to Cenozoic, yet seismic studies on the relationship between crust extension and lithospheric mantle deformation are scarce. Here we present a three dimensional radially anisotropic model of NCC derived from adjoint traveltime tomography to address this issue. We find a prominent low S-wave velocity anomaly at lithospheric mantle depths beneath the Taihang Mountains, which extends eastward with a gradually decreasing amplitude. The horizontally elongated low-velocity anomaly is also featured by a distinctive positive radial anisotropy (V_SH_ > V_SV_). Combining geodetic and other seismic measurements, we speculate the presence of a horizontal mantle flow beneath central and eastern NCC, which led to the extension of the overlying crust. We suggest that the rollback of Western Pacific slab likely played a pivotal role in generating the horizontal mantle flow at lithospheric depth beneath the central and eastern NCC.

## Introduction

**Figure 1:**
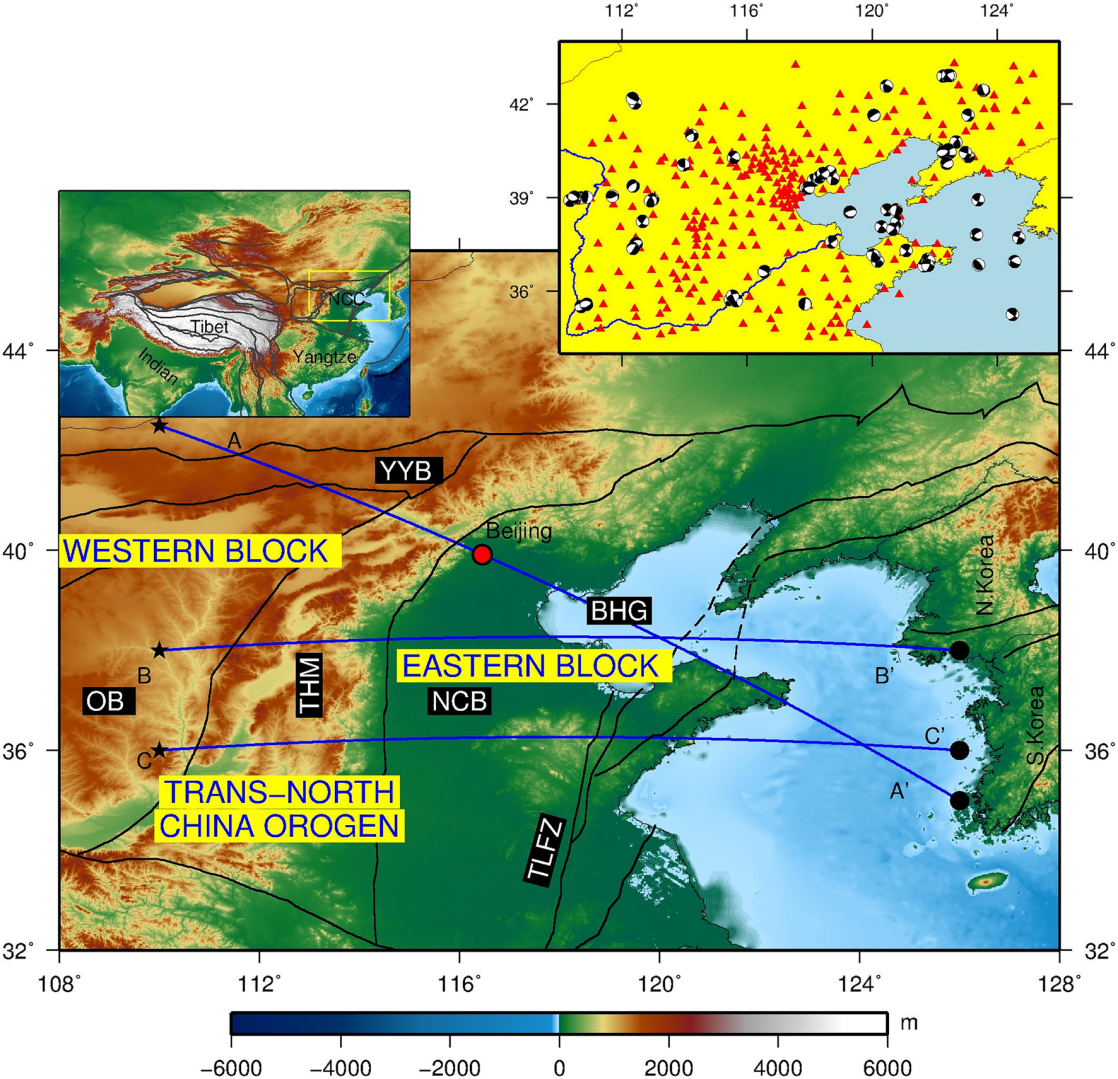
Geological and topographic map of the North China Craton. Blue lines A–A′, B–B′ and C–C′ indicate the location of three vertical profiles. YYB: Yinshan-Yanshan Block; OB: Ordos Block; THM: Taihang Mountains; NCB: North China Basin; BHG: Bohai Gulf; TLFZ: Tan-Lu Fault Zone. The left inset shows the location of the NCC in East Asia; the right inset shows the focal mechanisms of earthquakes (beach balls) and seismic stations (red triangles) in the study. The map in the figure was generated by the Generic Mapping Tools package^115^ (GMT-4.5.9, https://www.generic-mapping-tools.org/).

The thick lithosphere with low density and water content enables cratons to float on the asthenosphere and remain stable as indicated by lack of large-scale crustal deformation and magmatism^1,2^. However, since the Late Mesozoic, the eastern block of NCC has frequently experienced magmatic activities and multiple intense crustal deformations, which suggests that the original stable craton has been modified and destroyed^3^. This geological phenomenon challenges the classical plate tectonics theory (e.g., McKenzie and Parker^4^; Le Pichon^5^). Due to the coexistence of the highly extended eastern block and the stable western block (Fig. 1), the NCC offers a unique opportunity to address the fundamental framework of the evolution of cratons^6^.

Direct evidence for a complete or partial replacement of the lithospheric mantle beneath NCC comes from petrologic and geochemical studies. Geothermal studies of diamonds and mantle xenoliths indicate that the thickness of the lithosphere in the eastern NCC was approximately 200 km in the Paleozoic era (e.g., Griffin et al.^7,8^; Menzies et al.^9^). Re-Os isotope dating shows that these xenoliths are remnants of Archean lithospheric mantle (e.g., Gao et al.^10^; Wu et al.^11^; Zhang et al.^12^). However, geochemical studies of basalts collected from the area indicate that the highly depleted Paleozoic lithosphere was somehow replaced by fertile peridotites in the Cenozoic, and seismic studies suggest that the present-day lithospheric thickness of the eastern NCC is approximately 80 ~ 120 km^13,14^. Previous studies showed that heat flow had increased from ~ 40 Mw/m2 in the Paleozoic to 80 Mw/m2 in the Cenozoic (e.g., Nehru and Reddy^15^; Rao et al.^16^; Karmalkar et al.^17^). The high heat flow values are close to those observed in rift zones and other modern active continental areas. Previous studies suggest that the eastern NCC has undergone a stage of remobilization and has experienced intense extension with lithospheric root destruction^18,19^.

In contrast to the consensus on the replacement of mantle lithosphere beneath the eastern NCC, debates continue regarding the mechanism and dynamic processes leading to the destruction of the cratonic root. At present, there are two main types of models, namely, delamination (e.g., Gao et al.^20^; Wu et al.^21^; Xu et al.^22^; Deng et al.^23^) and thermal-chemical erosion (e.g., Zheng et al.^24^; Menzies et al.^25^; Zhai et al.^26^), which correspond to a top-down physical process and a bottom-up chemical–mechanical process, respectively. In terms of delamination, several different interpretations have been proposed, such as “local multiple delamination”, “lower crustal delamination” and “whole lithosphere delamination” (e.g., Jull and Kelemen^27^; Elkins-Tanton^28^; Xu et al.^29^). Multiple types of erosion models have also been suggested, including uniform erosion, mantle replacement and peridotite-melt interactions(e.g. Zheng et al.^24^; Menzies et al.^25^). Similarly, various plausible proposals have been presented for the dynamic processes that led to delamination or erosion, such as continental collision between the Yangtze Craton and the NCC^30^, intraplate asthenospheric upwelling^31^, India-Eurasia collision^32^, and paleo-Pacific Plate subduction^33,34^.

The basement of the NCC can be divided into three first-order tectonic units: the eastern NCC block, the western NCC block, and the Trans-North China Orogen in the middle, which formed in the Paleoproterozoic (about 1.85 Ga) due to the collision of the eastern and western blocks^35^. More specifically, the NCC consists of several subsidiary terranes, such as Yinshan-Yanshan block in the north, Ordos block in the west, Taihang Mountains in the middle, North China Basin, Bohai gulf and Tan-Lu Fault Zone in the East (Fig. 1). Previous models inverted from classical ray-based tomography (e.g., Tian et al.^36^; Guo et al.^37^; Wang et al.^38^) or finite-frequency tomography (Xu et al.^39^) captured stable large-scale patterns of NCC, such as the high wave speed and thick cratonic lithosphere of the Ordos block. These models differ in detail due to significant differences in ray-tracing methods, seismic phases selection and data weighting, but all models suffer from inaccurate crustal correction when involving joint inversion structures of crust and upper mantle and inherent theoretical limitations of approximate, asymptotic methods. Besides, complicated three dimensional (3D) geological structures of NCC result in severely distorted waveforms, which cannot be predicted using the imperfect one-dimensional (1D) reference model widely used in traditional tomographic inversions. Adjoint tomography completely eliminates these limitations and has demonstrated significant improvements in image accuracy of the Earth’s interior as compared to the results obtained from traditional ray-based tomography (e.g., Tape et al.^40^; Fichtner et al.^41^; Zhu et al.^42,43^; Chen et al.^44–46^; Tao et al.^47^; Dong et al.^48^; Huang et al.^49^). Taking advantage of dense seismic arrays deployed in NCC and adjoint tomography techniques, we present a seismic model named NCRA2021 (North China Radially Anisotropic model 2021), which reveals new details of low-velocity distribution and radially anisotropic pattern that further constrain lithospheric structure and infer deformation of the NCC.

## Data and waveform tomography

### Data

To obtain high-resolution 3D lithospheric images beneath the NCC, we collected waveform data from 67 earthquakes, and recorded by 270 broadband stations of the permanent seismic network of the China Earthquake Administration^50^ (Fig. 1). The earthquakes have a moment magnitude of Mw4.0-6.0 that occurred within the study area. These moderate size earthquakes are large enough to generate high signal-to-noise ratio (SNR) waveform data at the 270 stations, yet they are still not too large to violate the point source assumption employed in our inversion. Before inversion, we preprocessed these seismic data by first eliminating the instrument responses from the original data and then filtering data with a bandpass filter of 0.01–0.125 Hz. We also inverted the moment tensor of each earthquake using the generalized cut-and-paste (gCAP) method^51^.

### Adjoint traveltime tomography

Adjoint tomography has been developed in recent years to obtain high-resolution seismic images of the Earth’s interior by minimizing the misfit between predicted and observed waveform data (e.g., Fichtner et al.^41^; Lailly^52^; Liu and Tromp^53^; Tape et al.^54^). An essential step in adjoint tomography is calculating the Fréchet derivatives with adjoint technique (Liu and Tromp^53^; Tromp et al.^55^) by convoluting the forward wavefield generated by seismic events and the adjoint wavefield generated by the time-reversal adjoint source functions at the receivers (e.g., Tarantola et al.^56^; Tromp et al.^55^). Although the adjoint method was initially applied to “full waveform inversion” in exploration seismology (e.g., Gauthier et al.^57^; Mora^58^; Tarantola et al.^56^; Pratt et al.^59^; Brossier et al.^60^; Virieux and Operto^61^), it has been combined with the finite-frequency theory for seismic adjoint tomography (e.g., Marquering et al.^62,63^; Dahlen et al.^64^; Hung et al.^65^; Montelli et al.^66^). Adjoint tomography utilizes iterative strategy to invert subsurface structures based on more realistic 3D heterogeneous model and full seismic wavefield simulation with highly accurate numerical methods, such as the spectral element method^67,68^. Currently, local optimization based on gradient descent is widely adopted to solve the adjoint tomography problem, which requires an initial model kinematically compatible with the observed data within half a wavelength to prevent cycle skipping problems^61^. Luo and Schuster^69^ attempted to extract traveltime residuals from the cross-correlation of predictions and observations to construct misfit functions; this method is insensitive to cycle-skipping problems and increases the probability of iterations converging to a global minimum. Therefore, we apply adjoint traveltime tomography to obtain 3D seismic velocity structure of lithosphere and upper mantle beneath the NCC.

Our inversion involves the following three variables: compressional wave velocity (V_C_),

vertically polarized S-wave velocity (V_SV_) and horizontally polarized S-wave (V_SH_) velocity. The objective function, δχ, can be written in the form of a volume integral^55,70,71^ as follow:

1 $$\delta \chi = \int {(K_{{V_{c} }} \delta \ln V_{c} + K_{{V_{{sv}} }} \delta \ln V_{{sv}} + K_{{V_{{sh}} }} \delta \ln V_{{sh}} )d^{3} x}$$

where $K_{{V_{c} }}$, $K_{{V_{{sv}} }}$ and $K_{{V_{{sh}} }}$ correspond to the Fréchet kernels of a compressional, SV and SH waves, respectively. We used spectral-element codes SES3D^72^ to simulate both the forward and adjoint wavefields, and then calculated the Fréchet derivatives of (model parameters) V_C_, V_SV_ and V_SH_.

For the 3D complex North China lithosphere model, using full band seismic data may make inversion trapping into local minima. Therefore, we adopted a multiscale strategy and conducted the inversion in two frequency bands (8–50 s and 20–100 s) to mitigate cycle skipping. The inversion results of the low frequency band (20–100 s) were used as the initial velocity model of the next inversion with high frequency (8–50 s) data. We employed the FWEA18 (Full Waveform Inversion of East Asia in 2018^47^) as our initial model. In order to minimize numerical dispersion, we paid special attention in mesh generation, i.e., the length of each spectral element was ensured to be less than half of minimum wavelength. The open-source package FLEXWIN^73^ was used to automatically select time windows between paired synthetic and observed waveforms.

The overall cross-correlation traveltime misfit function ***χ(m)*** for the current Earth model ***m ***of all the selected windows is written as

2 $$\chi (m) = \frac{1}{2}\frac{1}{{N_{\omega } }}\sum\limits_{{e = 1}}^{E} {\sum\limits_{{i = 1}}^{{N_{\omega }^{s} }} {\left[ {T_{i}^{{obs}} - T_{i} (m)} \right]^{2} } }$$

where $N_{\omega }^{s}$ denotes the number of time windows of earthquake $e$, E indicates the total number of seismic events, and $N_{\omega } = \sum\nolimits_{{e = 1}}^{E} {N_{\omega }^{s} }$ denotes all the selected time windows. The adjoint source of the corresponding time window is calculated based on Tromp et al.^55^:

3 $$f^{ * } (t) = - [T^{{obs}} - T(m)]\frac{1}{N}\partial _{t} s(T - t,m)\delta (x - x_{r} )$$

where $s$ denotes displacement. $N$ is a normalization factor given by

4 $$N = \int_{0}^{T} {\user2{s}(t,\user2{m})} \partial _{t}^{2} \user2{s}(t,\user2{m})dt$$

The gradients of all events were summed together to obtain the total gradient that suggests the direction for the model update. The gradient-based method was used for the inversion, and the model of the previous iteration was used as the initial model for the next iteration. We terminated the inversion process after 15 iterations once the reduction of the misfit residual becomes insignificant. After inversion, the mean values of traveltime shifts were significantly reduced. For the frequency bands of 20–100 s, the mean value changed from  − 0.6 to  − 0.3 s; for the frequency bands of 8–50 s, the mean value decreased from 0.36 to  − 0.01 s (Fig. S1).

### Model assessment

Due to the high cost of computational resource required by adjoint traveltime tomography, performing traditional “checkerboard” tests to assess resolution is almost unrealistic as it requires the same amount of computing as an actual structural inversion^43^. Therefore, we used the point-spreading-function test to assess local resolution (e.g., Fichtner and Trampert^74,75^; Chen et al.^45^). To do so, we first placed a 5% low-Vsv zone at the center of the study area at a depth of 20 km (Fig. S2) and computed Hessian kernels for the parameters V_C_, V_SH_, and V_SV_ (Fig. S2b–d). Although the results deviated slightly from the perturbation pattern, the main features were recovered and the tradeoffs between V_SH_ and V_SV_ (or V_C_ and V_SV_) were almost negligible (Fig. S2). We also conducted similar point-spreading-function resolution tests at depths of 60 and 100 km (Figs. S3, S4), and the results indicated that the main features of the perturbations could be recovered by our dataset. To further illustrate that our model can resolve 3D variations of lithospheric mantle in specific region (e.g., Taihang Mountains), a vertical point-spreading-function test along 39° N at depths between 85–115 km was carried out and the result showed that our model is reliable (Fig. S5).

Previous ray-based traveltime tomographic results have revealed many characteristic structures in the upper mantle beneath different geological units of the NCC (e.g., Huang & Zhao^76,77^; Zhao et al.^78^; Wang et al.^38^). Compared with previous models (e.g., Tian et al.^36^), NCRA2021 depicts a more continuous high-velocity variation of crystalline crust of Bohai gulf and a clearer and more coherent low-velocity lithospheric mantle beneath central and eastern NCC. The patterns of high and low velocity regions in NCRA2021 are roughly consistent with the images of finite-frequency tomography (Xu et al.^79^). However, the amplitudes of the low S-wave velocity anomalies beneath the Taihang and Yanshan mountains are much larger.

## Results and discussions

### Map views of V_SV_ anomalies and radial anisotropy

**Figure 2:**
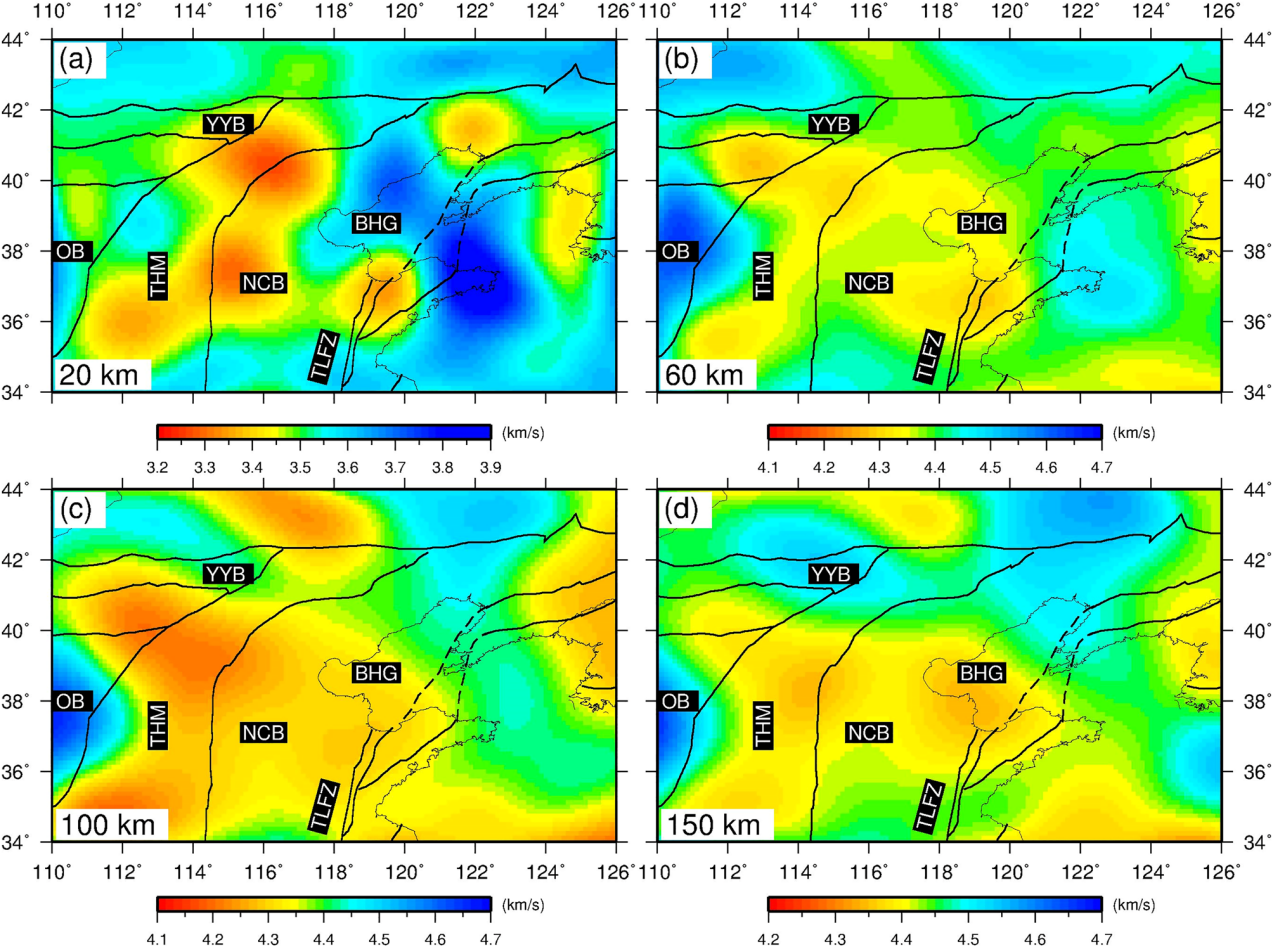
Map views of Sv-wave velocity at four depths. (**a**) 20 km, (**b**) 60 km, (**c**) 100 km and (**d**) 150 km. The map in the figure was generated by the Generic Mapping Tools package^115^ (GMT-4.5.9, https://www.generic-mapping-tools.org/).

We present a series of horizontal maps of SV-wave speed to illustrate the lithospheric heterogeneity beneath the NCC (Fig. 2). At a depth of 20 km, the Bohai gulf shows clear high shear wave velocity that further extends to the south of North China Basin, in contrast to eastern central tectonic belt and western North China Basin, which are dominated by a large-scale low velocity anomaly. The western part of the central tectonic belt appears to be penetrated by a high velocity anomaly underlying the Ordos block (Fig. 2a). At a depth of 60 km, both low velocity anomalies expands to a greater area including the Bohai gulf. On the other hand, the high-velocity anomaly beneath the Ordos block also extends to a broader region, including a large portion of the central tectonic belt (Fig. 2b). Between depths of 100 and 150 km (Fig. 2c, d), the study area is dominated by the widespread low velocity anomaly with a high amplitude.

**Figure 3:**
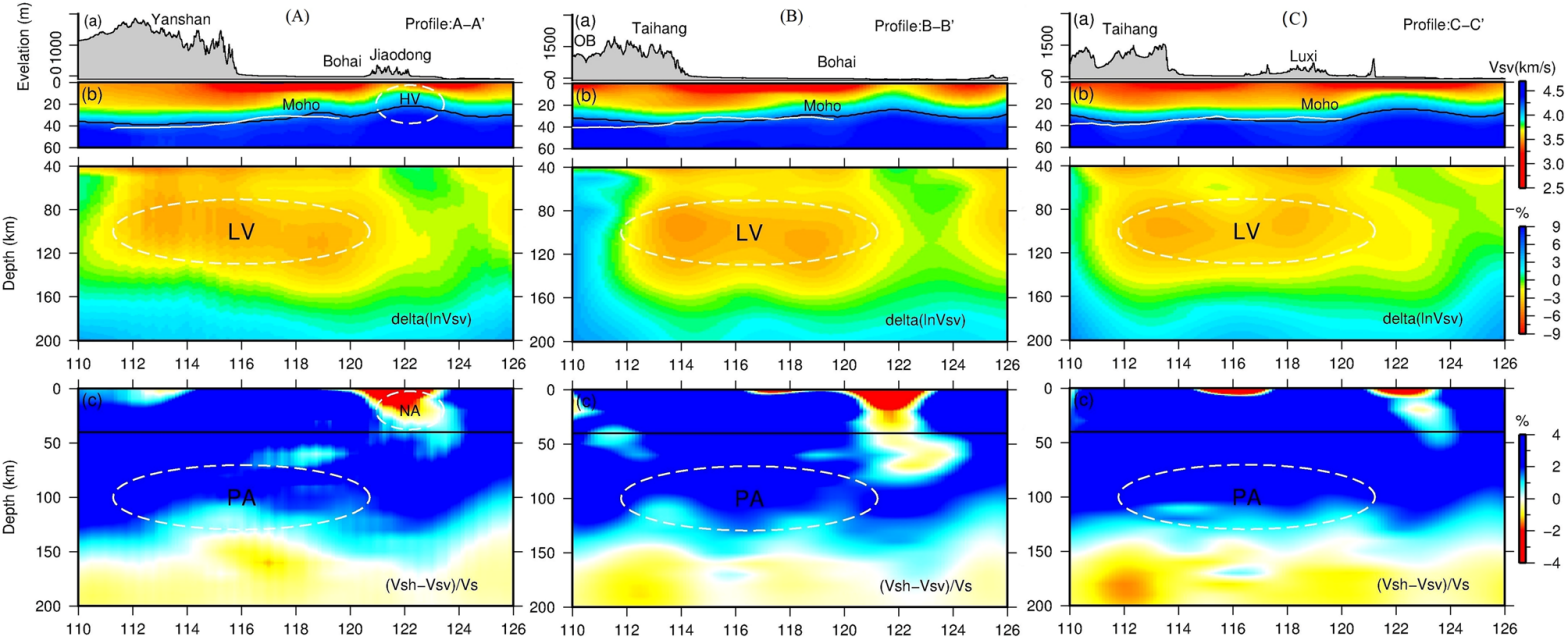
Three vertical cross sections with surface elevation, velocity anomaly and radial anisotropy along profile A–A′ (**A**), B-B′ (**B**) and C–C′ (**C**). Surface altitude (top, a), Sv-wave velocity anomalies (middle, b), and radial anisotropy (V_SH_-V_SV_)/V_S_ (bottom, c). For sub-graph b, absolute velocity is adopted for the crust (top 60 km), and velocity perturbation is adopted in upper mantle (40–200 km) with reference model: PREM (Dziewonski and Anderson 1981). White solid line denotes the depth of Moho obtained from receiver function (Chen 2009; Zheng et al. 2014); black solid line denotes the depth of Moho derived from reference shear wave velocity of 4.2 km/s. LV: low velocity; HV: high velocity; NA: negative anisotropy; PA: positive anisotropy. The map in the figure was generated by the Generic Mapping Tools package^115^ (GMT-4.5.9, https://www.generic-mapping-tools.org/).

**Figure 4:**
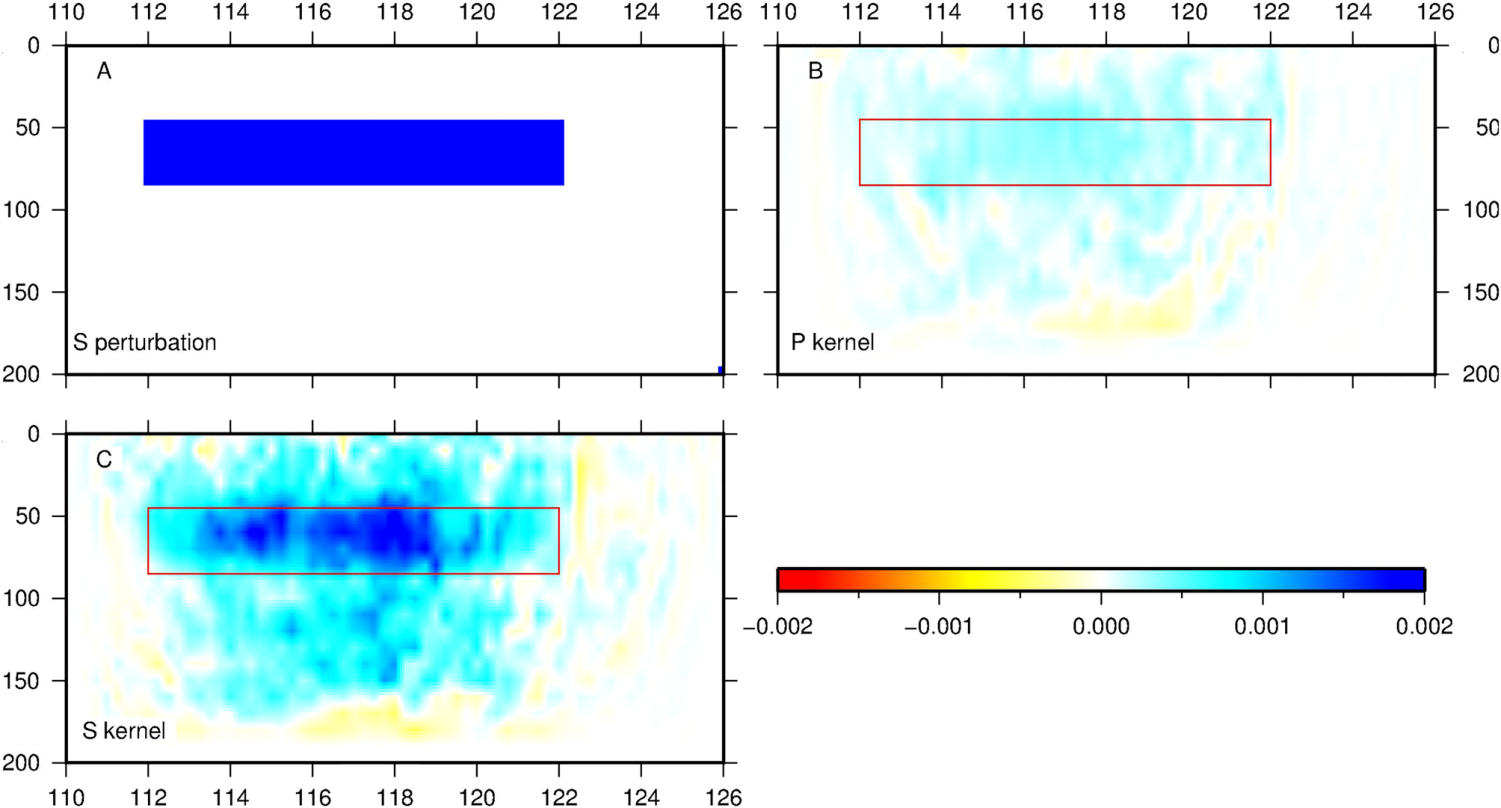
Sensitivity test of Shear wave in the uppermost mantle along 37° N between the depth of 45 km and 85 km. (**a**) input high Shear velocity perturbation, (**b**) sensitivity kernel of P wave, (**c**) sensitivity kernel of S wave. The unit of the color bar is 1 × 10^–10^ s^2^m^-4^. The map in the figure was generated by the Generic Mapping Tools package^115^ (GMT-4.5.9, https://www.generic-mapping-tools.org/).

Three vertical sections across the eastern and western parts of North China, marked as A–A′, B–B′, and C–C′ in Fig. 1, further illustrate the lateral variations of lithospheric structure across the NCC. In each section, the top, middle and bottom plots show, respectively, the absolute V_SV_ of the top 60 km of the lithosphere, velocity perturbations (*dlnV*_*SV*_) in the depth range of 40—200 km, and the radial anisotropy down to 200 km depth. We employed the 1-D PREM model^80^ in computing velocity perturbations. Profile A-A’ runs across the YYB, BGH and Jiaodong with significant variations in elevation (Fig. 3). We defined the Moho as the isochron velocity of 4.2 km/s ($\nu _{{\mathbf{s}}}^{{{\text{iso}}}} = 2\nu _{{{\mathbf{SH}}}} /3 + \nu _{{{\mathbf{SV}}}} /3$, black solid line in Fig. 3A(a)). Crustal thickness decreases gradually from west to east, which is consistent with the results of receiver function studies (solid white line in Fig. 3A(a), e.g., Chen^12,13^). The shallowest Moho lies beneath the Jiaodong area, which is also featured by a strong negative radial anisotropy (V_SV_ > V_SH_) (read area labeled as NA in Fig. 3A(c)). This may suggest that area has experienced a unique tectonic event dominated by vertical deformation. The most prominent velocity structure shown in the perturbation map is the broad and strong low velocity anomaly beneath the central and eastern NCC (Fig. 3A(b)), suggesting that the high velocity cratonic lithosphere has been replaced. In order to whether the high velocity cratonic keel was completely or partly removed, we conducted a numerical experiment. We added a thin high velocity layer that extends from 112° E to 122° E in the depth range of 45–85 km, and our results indicated our such a high velocity layer would be easily resolved by our inversion (Fig. 4). Therefore, we concluded that the observed large depth extent of the low velocity structure is robust and the cratonic lithosphere here was completely replaced. The other two profiles (B–B′ and C–C′) also reveal approximately similar patterns with profile A–A′ (Fig. 3B and C). We made a detailed comparison between NCRA2021 and FWEA18 along profile A–A′, which demonstrates that our model has significantly improved the initial model FWEA18^47^. In Fig. S6, the depth of the Moho given by the NCRA2021 model is closer to that from receiver functions^12,13^. The Moho depth of FWEA18 exhibits an anomalous discontinuity beneath Yanshan, which is contrary to the result of receiver function results. Another noteworthy issue is that the NCRA2021 model shows that the lower crust is significantly uplifted in the Jiaodong area, reflecting the large-scale magmatic activity and extensional structural deformation experienced in the area during the Early Cretaceous. This may be the reason for the wide-spread gold deposits in the Jiaodong area^81^.

### Horizontal mantle flow beneath the NCC

Radial anisotropy of upper mantle may be caused by the subhorizontal lattice-preferred orientation of olivine and its association with low-velocity zones suggests olivine alignment due to flow in the upper mantle of relatively low mechanical strength (e.g., Gao et al.^82^; Savage^83^; Karato et al.^84^). Petrophysical experiments have illustrated that a small amount of melt can cause a significant decrease in seismic wave velocity. For example, Sato and Sacks^85^ found that as long as 5% of the melt in the mantle peridotite exists, it can cause the seismic wave velocity to drop by about 5%. Our model shows widespread low-velocity zones in the lithospheric mantle beneath central and eastern blocks and prominent low S-wave perturbations (exceed 5%) under mountains, which might indicate the presence of a certain amount of melt. These weak material regions are dominated by strongly positive radial anisotropy (V_SH_ > V_SV_), indicating that they are subjected by intense horizontal strain, which makes them possible to flow on a geological time scale. According to our model, the shear wave traveltime in a 120 km thick lithosphere is about 26.7 s (the average S-wave velocity is ~ 4.5 km/s) associated with the 4% anisotropy, then the traveltime delay between the fast and low S-wave is ~ 1.1 s. The average time delay of SKS wave splitting in the central-eastern part of North China is ~ 1.0 s with the NW–SE fast S-wave direction (Liu et al.^86^; Wang et al.^87^; Zhu and Ma^88^), which support our claim of horizontal mantle flow. Moreover, GPS measurements show that the current crust of the North China region is moving in the direction of NW–SE (Wang et al.^89^), which is also the direction of subduction and retreat of the Western Pacific slab. Therefore, the reasonable direction of plausible mantle flow should be NW–SE.

High magnesium andesites, dacites and adakites in the NCC are considered to be products of the interactions between partially melted eclogites and mantle rocks^20^. One explanation is that crustal materials delaminated into the mantle. Based on the above tomographic images, the large low-velocity regions under the Taihang and Yanshan Mountains might be caused by lower crustal thicken and delamination. Due to subduction of the western Pacific Plate, the crust beneath the Taihang and Yanshan Mountains thickened, which promoted the transformation of the mafic lower crust into eclogites^10,20^. Since the eclogite is denser than the ambient mantle, it will sink into the mantle due to gravitational instability (e.g., Lustrino et al.^90^; Bédard et al.^91^; Arndt and Goldstein^92^). Admittedly, crustal delamination would be much harder to occur within a craton than in an arc setting, so there may be other important factors leading to lithospheric mantle instability. The mantle transition zone (denotes as MTZ) is composed of wadsleyite and ringwoodite, which can hold more water than the peridotite mantle, and the water content of the MTZ in eastern China is at least 0.5–1 wt% (Kelbert et al.^93^). Geodynamic simulations indicate that when the subducted slab interacts with the wet MTZ, the water in the MTZ will be squeezed out, and part of the water entering the upper mantle would promote partial melting and form the low-velocity zone^94^. These molten mantle regions will promote metasomatism and lead to refertilization and rejuvenation of the lithospheric mantle^95^. These processes could increase the density of the lithospheric mantle and weaken it, which is conducive to the process of delamination (Fig. 4). Lithospheric delamination would cause upwelling of magmatic materials, and these partially melted weak materials may flow horizontally under long-term extension.

The studies of receiver function reveal that the depth of 410 km discontinuity in central NCC sinks about 10 km^96^, which may be caused by the temperature increase at 410 km discontinuity owing to the extruded water and soluble radioactive elements from MTZ^94^. Higher temperature increases the pressure required for phase transformation form α-olivine to β-wadsleyite and deepen the depth of phase transformation^97^. One of the signs of NCC activity is extensive magmatism. During the period from 200 ~ 140 Ma, the magmatic activity migrated inland from the trench, and after 140 Ma, the magmatic activity migrated continuously southeastward^98^. These two stages of magmatic activity represent the response of the western Pacific Plate to subduction advance and retreat, respectively^39^. These changes in subduction represent an external factor and the dynamic background for the destruction of the NCC. The internal factor for the destruction of the NCC was the exchange of material and energy between the deep and shallow levels^99^. In addition to magmatic activity, the timing of ductile extensional metamorphic core complexes in North China tends to become younger from northwest to southeast^100^. Magmatism and metamorphic core complexes migrated in the same direction as the horizontal mantle flow mentioned above and can be interpreted as resulting from horizontal mantle flow.

### Geodynamic mechanism of the NCC destruction

From the perspective of continental dynamics, there are two possible reasons for the destruction of the NCC: (1) the subduction of an oceanic plate (e.g., Zhu et al.^19^) and (2) the dynamic action of the deep mantle (e.g. Wilde et al.^31^). Simulation of the stability of the continental lithosphere shows that the thermal erosion at the bottom of typical cratonic lithospheric is limited (King et al.^101^; Hieronymus et al.^102^). Even if it is directly located above a mantle plume, a cratonic lithospheric mantle root would require more than 200 Ma to be significantly eroded. In addition, numerical simulations of geodynamics also illustrate the crucial role of plate motion in continental drift (Yoshida^103^). Li et al.^104^ indicated that the negative buoyancy from lithospheric thickening during orogenesis could cause delamination when the reference density of the lithospheric mantle is not lower than that of the asthenosphere. However, if the reference density of the lithospheric mantle is less than that of the asthenosphere, additional contributing factors, such as lower crust eclogitization, are required for delamination. Hu et al.^105^ suggested that significant modifications of the cratonic lithosphere in South America and Africa reflect permanent increase in lithospheric buoyancy due to plume-triggered delamination of deep lithospheric roots during the Late Cretaceous period and early Cenozoic era. Note that their conclusions are based on simulations of passive continental margin dynamics. However, the NCC has been affected by the subduction of the western Pacific Plate since the late Mesozoic, so its modification mechanism may be different. Zhu & Xu^106^ suggested that the cratonic lithosphere is severely hydrated and that non-steady mantle flow develops, resulting in metasomatism, melting and weakening of the lithosphere, which ultimately leads to lithospheric thinning and cratonic destruction.

**Figure 5:**
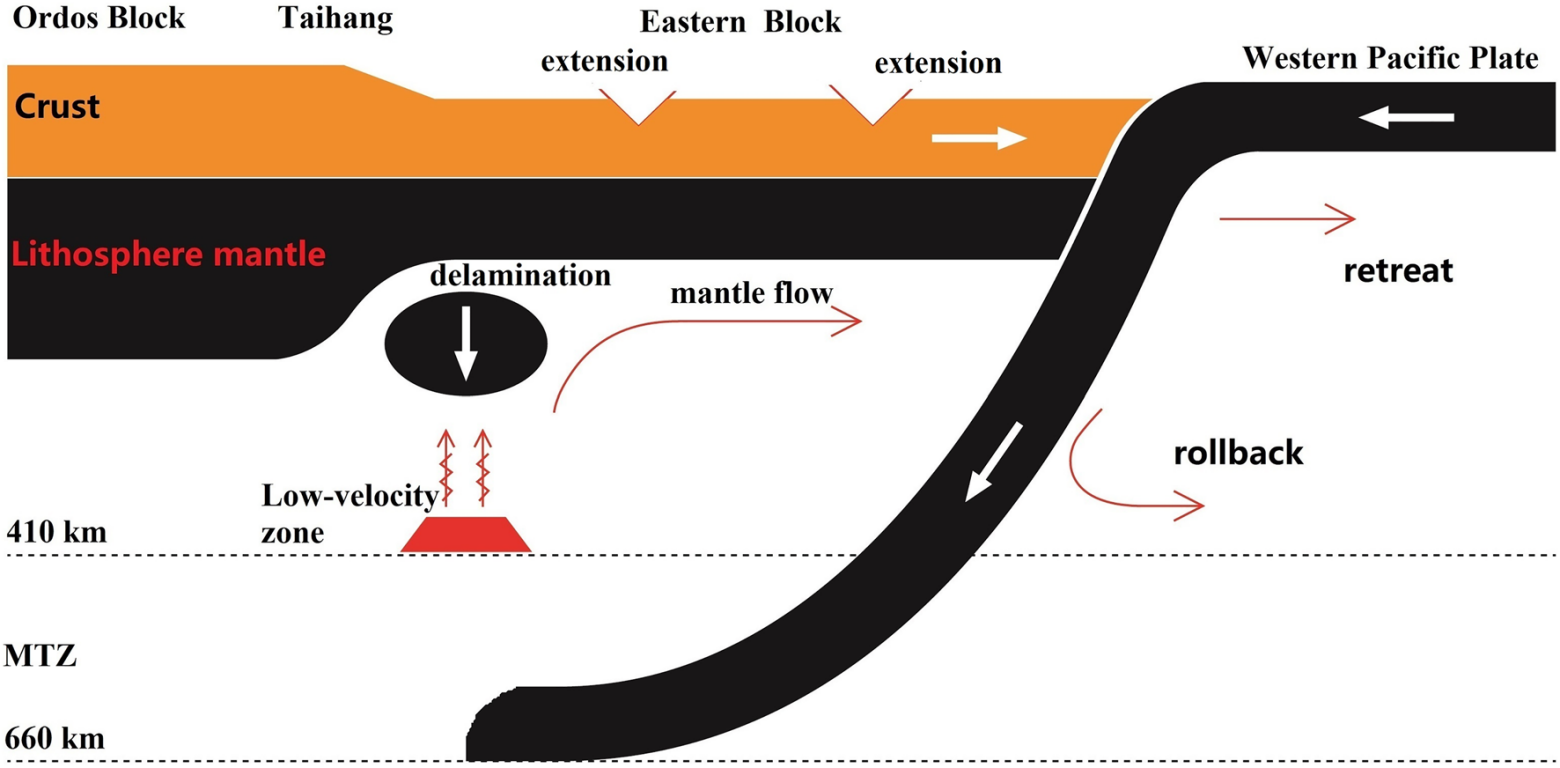
The subduction, rollback and retreat of the western Pacific Plate cause the delamination, horizontal mantle flow and continental crust extension of the NCC. The red trapezoidal area represents the low-velocity zone formed by the subducting slabs squeezing out the water in the mantle transition zone (denotes as MTZ); the water enters the upper mantle and causes partial melting. The black ellipse indicates the delamination of lithosphere due to gravity instability.

The existing observational data indicate that the subduction of the Pacific Plate under East Asia has played an important role in the destruction of the eastern NCC since the Mesozoic (Griffin et al.^8^; Wu et al.^21^; Ren et al.^107^). The subduction hanging wall of the Western Pacific lithosphere has undergone multiple stages of extension, coexisting island-arc volcanism and fore-arc extension of accretionary wedge since late Mesozoic (Zhu and Xu^106^). A convincing explanation is that the Western Pacific plate has experienced multiple processes of rollback and trench retreat (Yoshida^108^). The rollback of the oceanic slab induces the upwelling magma owing to the delamination (and/or other mechanisms) to flow horizontally, resulting in continental crust extension and large-scale magmatism in the eastern NCC (Fig. 5). The puzzle is that the current onset age of the Western Pacific subduction is 50–60 Ma (Moverly^109^; Taylor^110^), while the lithospheric thinning in NCC mainly occurred in the Mesozoic, probably before 110 Ma (Liu et al.^111^). Therefore, the Pacific plate lying flat in the MTZ is Cenozoic, not the source of Mesozoic lithospheric thinning in North China. A reasonable explanation is that the subduction of the western Pacific plate in Mesozoic led to the large-scale thinning of the lithosphere in North China, forming widespread low-velocity zones (LVZs) in lithospheric mantle. The dehydration of the Cenozoic western Pacific plate in the MTZ provides water (in the form of water-rich melt) for the LVZs of the lithospheric mantle (Niu^112,113^), maintaining the already formed LVZs. Plate reconstruction in northeastern Asia indicates that the western Pacific oceanic plate subducted westward under East Asia along Mudanjiang-Honshu Island during the Jurassic, and that the trench retreated to the Sikhote-Alin, North Shimanto, and South Shimanto zones from ca. 137–128 Ma, ca. 130–90 Ma, and ca. 60 Ma, respectively (Liu et al.^114^). These studies provide supports for the mechanism that weak lithospheric mantle materials flowed horizontally in response to the rollback of the western Pacific slab.

## Conclusions

The tectonic reanimation of the NCC indicates that the stable craton can also be modified and destroyed, and this special geological phenomenon is the product of continental lithosphere evolution under the subduction and rollback of the oceanic slab. A complete three-component dataset including body and surface waves is inverted together with periods ranging from 8–100 s to obtain the 3D radially anisotropic model of NCC lithosphere. Central and eastern NCC are characterized as prominent low S-wave velocity lithospheric mantle coupling with the primarily horizontal stress deformation, leading support to the hypothesis of horizontal mantle flow.

Our model favors the subduction of the western Pacific slab during the Mesozoic as the trigger for gravitationally unstable delamination. The subduction of oceanic slab makes the lower crust of the central NCC thickened; besides it squeezed out the water in the MTZ, resulting in partial melting and magmatism of the upper mantle. All of these make the stability of lithospheric mantle decrease and eventually lose stability. The upwelling magma owing to the delamination (and/or other mechanisms) was transported horizontally, and the driving force of this horizontal mantle flow may stem from the rollback of the western Pacific slab^81^. The dehydration reaction of the Cenozoic Western Pacific slab in the MTZ provided sufficient water source for the maintenance of the lithospheric LVZs.

## Supplementary Information

Supplementary Information.
