# Supplementary material for: Adjoint traveltime tomography unravels a scenario of horizontal mantle flow beneath the North China craton
Source: Sci Rep. 2021 Jun 15;11:12523. doi: 10.1038/s41598-021-92048-8 (PMC8206337; doi:10.1038/s41598-021-92048-8)
Supplement: Supplementary file 1 — Supplementary Information. [file 41598_2021_92048_MOESM1_ESM.docx]

**Adjoint traveltime tomography unravels a scenario of horizontal mantle flow beneath the North China craton**

Xingpeng Dong^1^, Dinghui Yang^1^*, Fenglin Niu^2,3^, Shaolin Liu^1, 4^, Ping Tong^4^

1. Department of Mathematical Sciences, Tsinghua University, Beijing 100084, China
2. Department of Earth, Environmental and Planetary Sciences, Rice University, Houston, Texas, USA
3. State Key Laboratory of Petroleum Resources and Prospecting, and Unconventional Petroleum Research Institute, China University of Petroleum at Beijing, Beijing, China.
4. Division of Mathematical Sciences, School of Physical and Mathematical Sciences & Asian School of the Environment, Nanyang Technological University, Singapore

Correspondence: Dinghui Yang (E-mail: ydh@mail.tsinghua.edu.cn)

**Contents of this file**

Figures S1 to S6


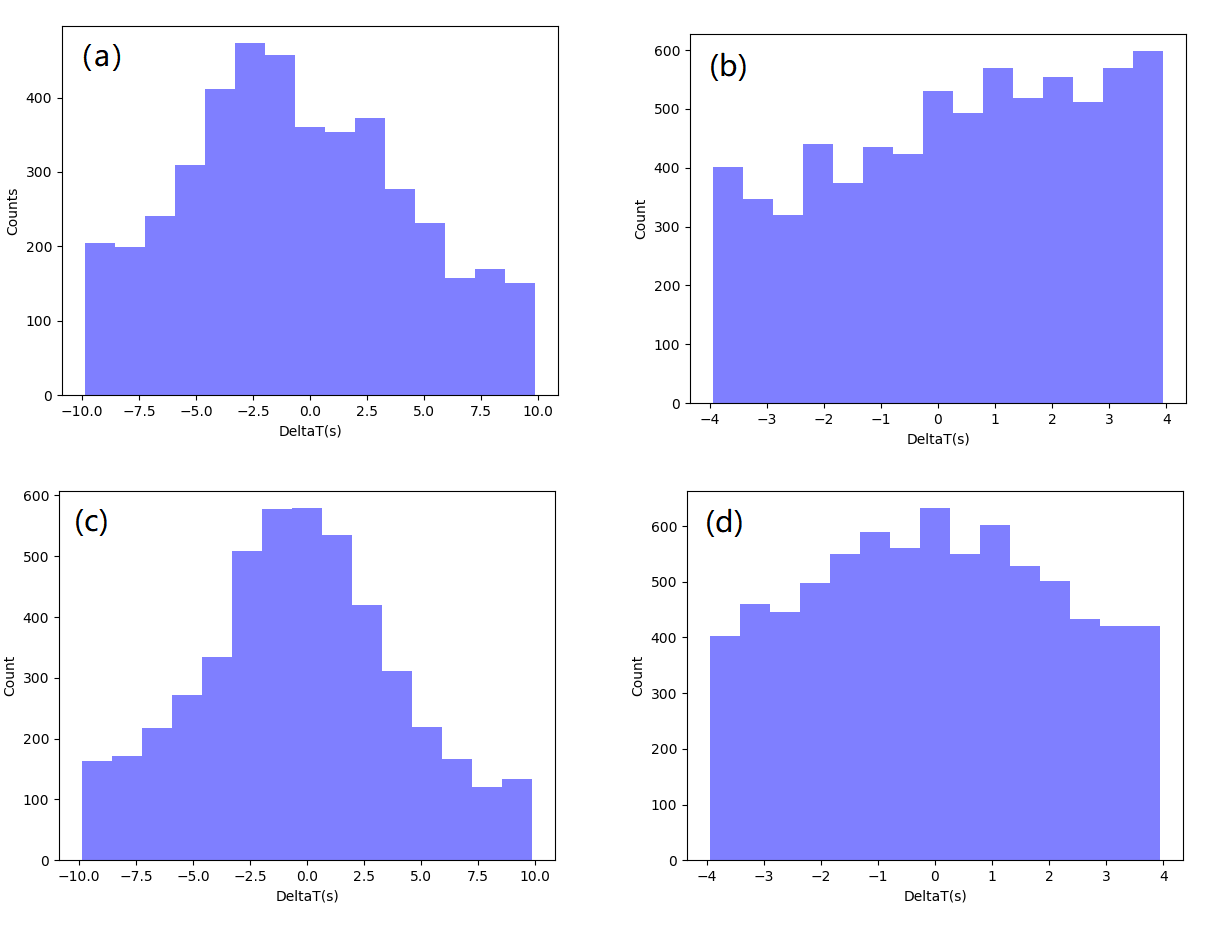


**Figure S1.** Histograms of the traveltime residuals before and after inversion in two frequency bands. (a) initial model of low-frequency band (20 – 100 s); (b) initial model of high-frequency band (8 – 50 s); (c) final model of low-frequency band (20 – 100 s); (b) finial model of high-frequency band (8 – 50 s).


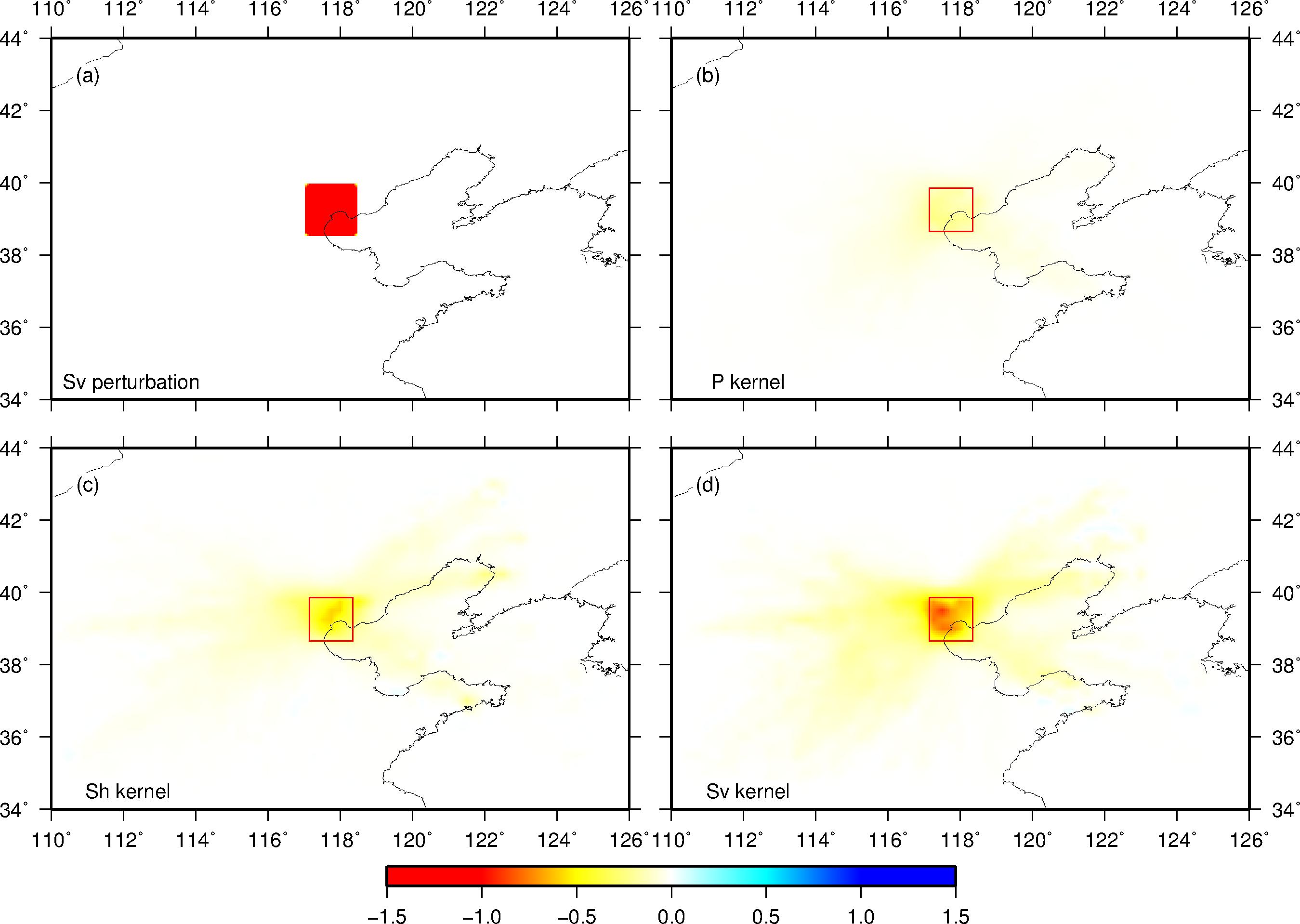


**Figure S2.** A point-spread function test of SV at the central of the study area at a depth of 20 km. (a) input low-SV velocity perturbation, (b) PSF of P wave velocity, (c) PSF of SH wave velocity, (d) PSF of SV wave velocity. The unit of the color bar is 1×10^-10^ s^2^m^-4^. The map in the figure was generated by the Generic Mapping Tools package^115^ (GMT-4.5.9, https://www.generic-mapping-tools.org/).


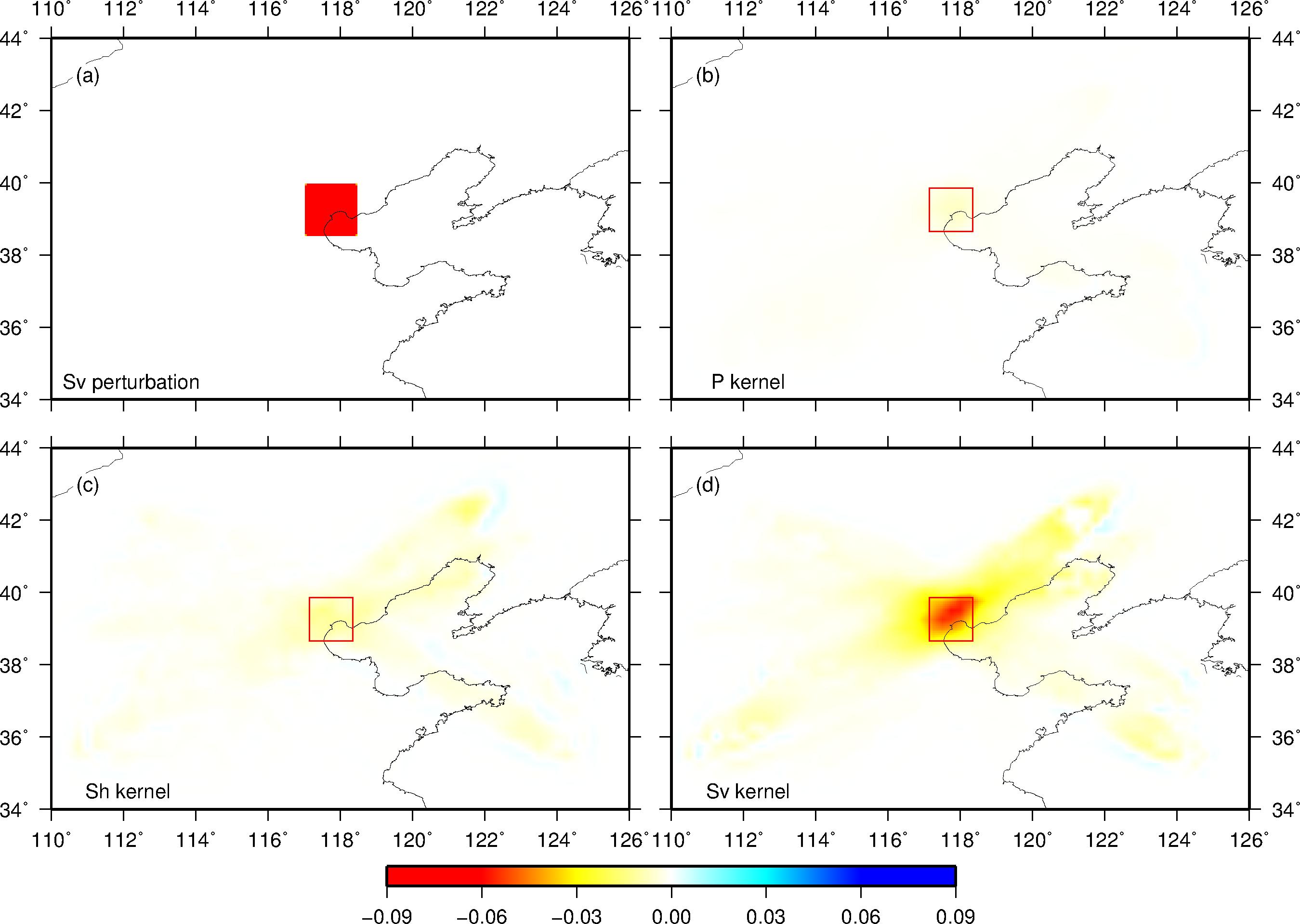


**Figure S3.** A point-spread function test of SV at the central of the study area at a depth of 60 km. (a) input low-SV velocity perturbation, (b) PSF of P wave velocity, (c) PSF of SH wave velocity, (d) PSF of SV wave velocity. The unit of the color bar is 1×10^-10^ s^2^m^-4^. The map in the figure was generated by the Generic Mapping Tools package^115^ (GMT-4.5.9, https://www.generic-mapping-tools.org/).


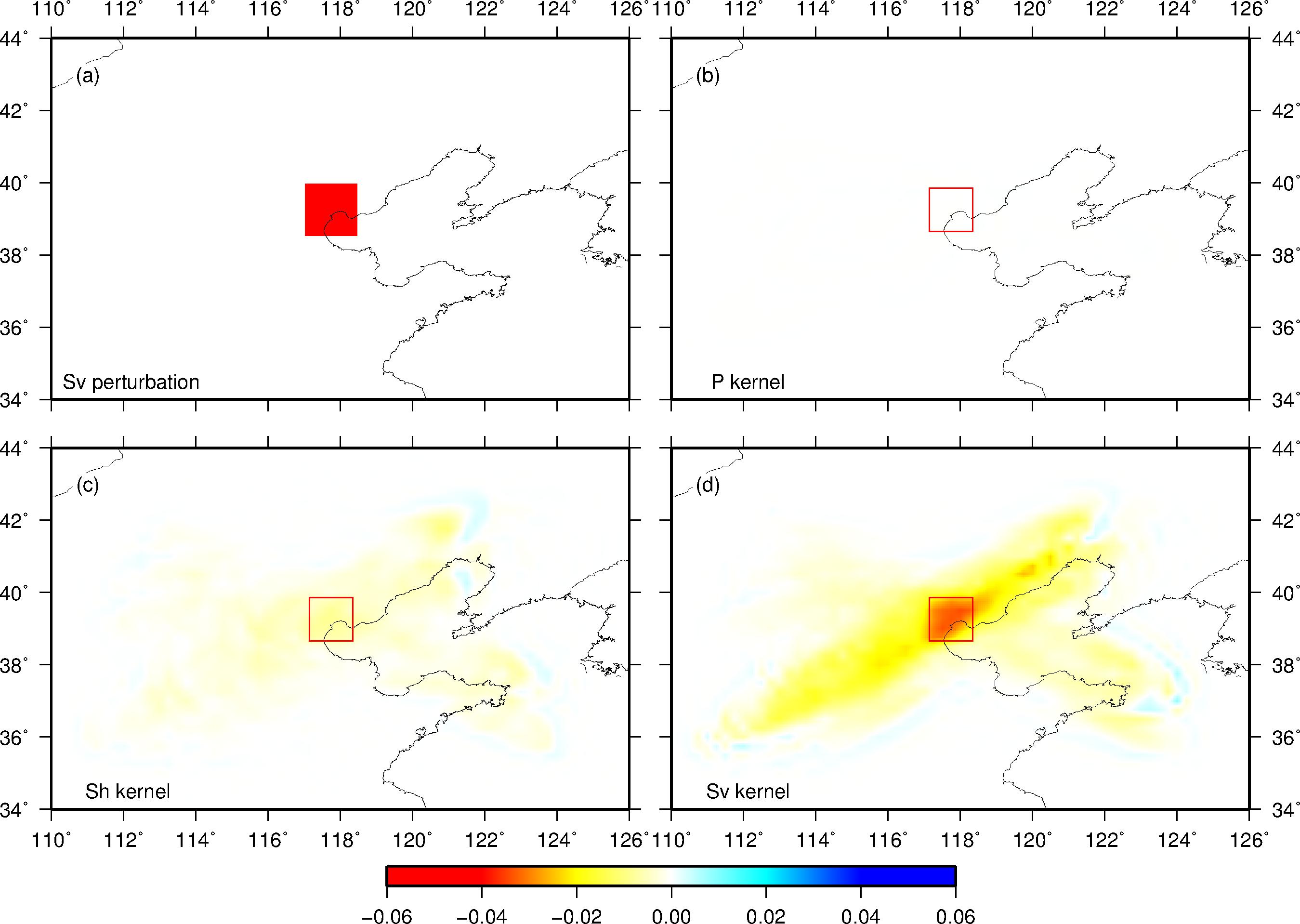


**Figure S4.** A point-spread function test of SV at the central of the study area at a depth of 100 km. (a) input low-SV velocity perturbation, (b) PSF of P wave velocity, (c) PSF of SH wave velocity, (d) PSF of SH wave velocity. The unit of the color bar is 1×10^-10^ s^2^m^-4^. The map in the figure was generated by the Generic Mapping Tools package^115^ (GMT-4.5.9, https://www.generic-mapping-tools.org/).


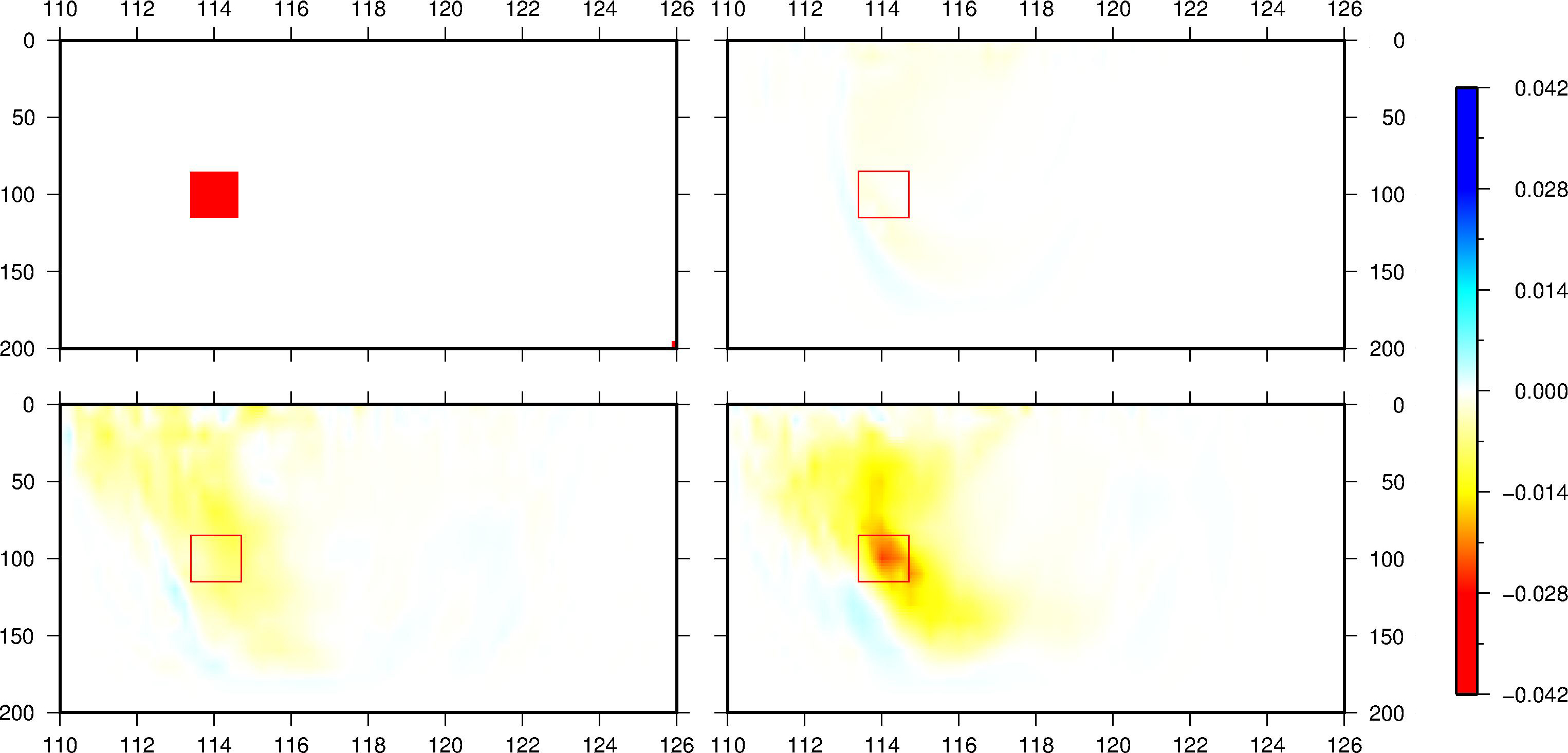


**Figure S5.** A point-spread function test of SV at the upper-left of the study area along 39° N. (a) input low-SV velocity perturbation, (b) PSF of P wave velocity, (c) PSF of SH wave velocity, (d) PSF of SV wave velocity. The unit of the color bar is 1×10^-10^ s^2^m^-4^. The map in the figure was generated by the Generic Mapping Tools package^115^ (GMT-4.5.9, https://www.generic-mapping-tools.org/).

**
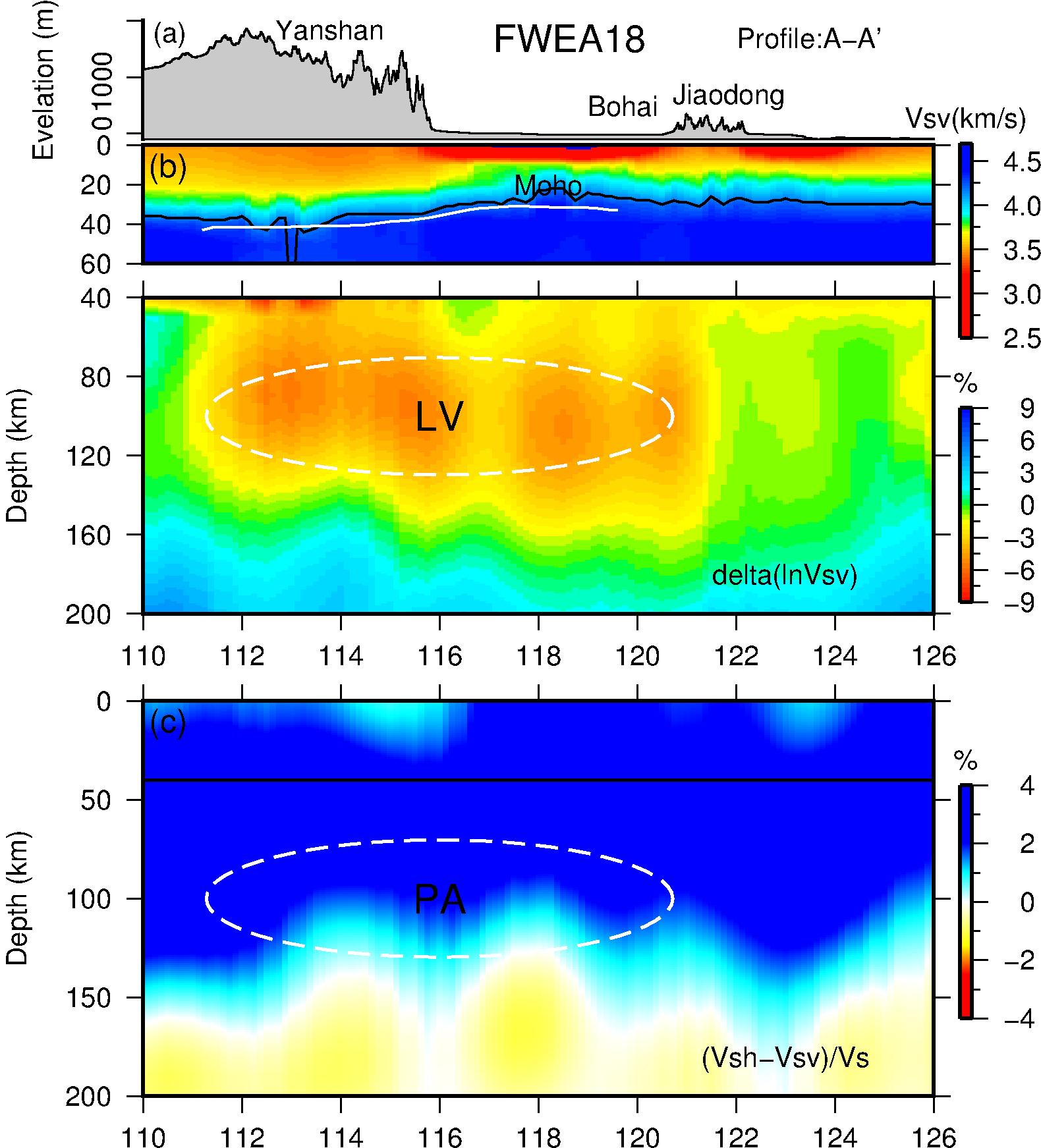
**

**
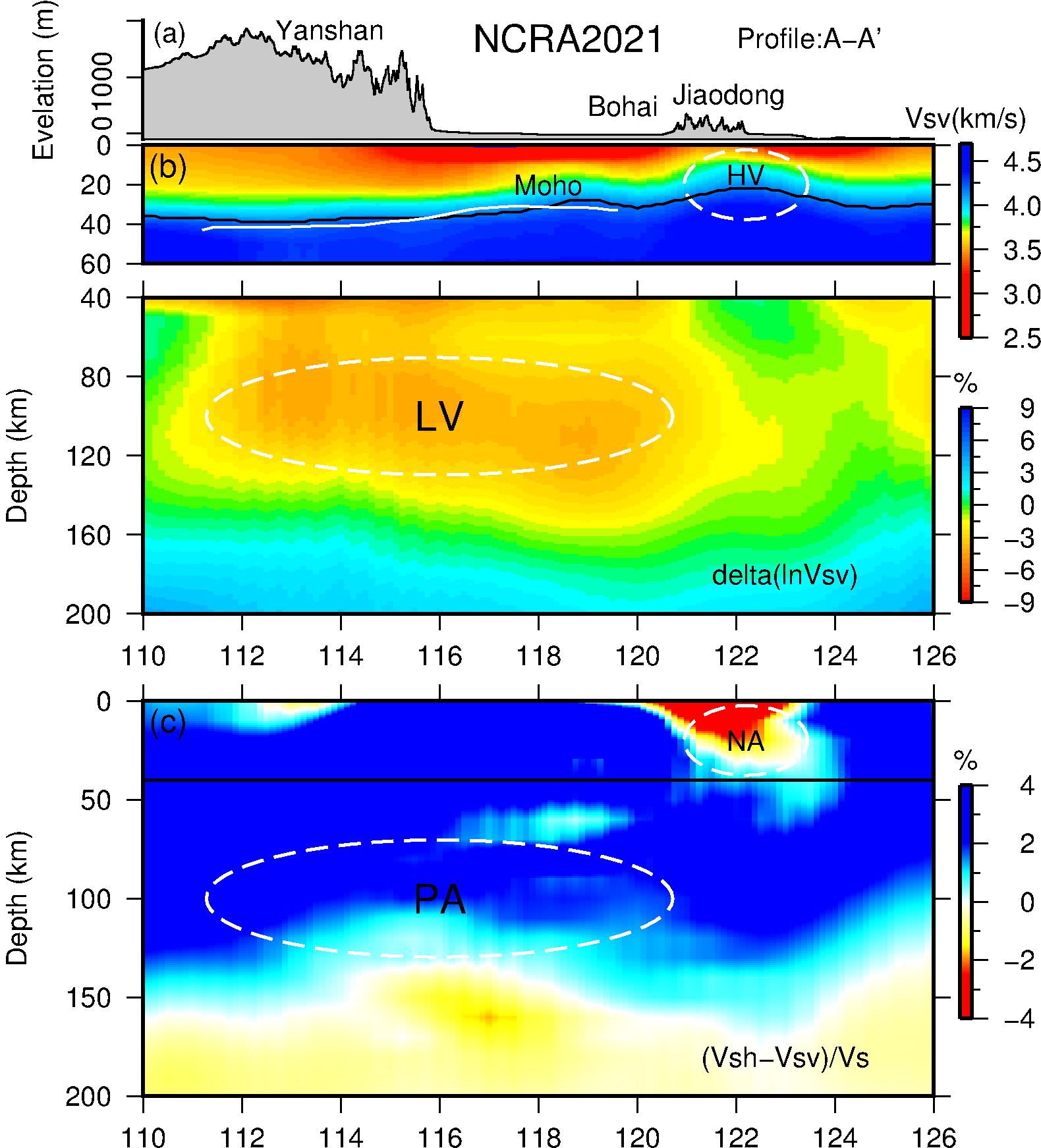
**

**Figure S6.** Three vertical cross sections with surface elevation, velocity anomaly and radial anisotropy along profile A-A’ for FWEA18 (top) and NCRA2021 (bottom). The map in the figure was generated by the Generic Mapping Tools package^115^ (GMT-4.5.9, https://www.generic-mapping-tools.org/).
